# From actinic keratosis to cutaneous squamous cell carcinoma: the key pathogenesis and treatments

**DOI:** 10.3389/fimmu.2025.1518633

**Published:** 2025-01-24

**Authors:** Zhenlin Li, Fangqi Lu, Fujin Zhou, Dekun Song, Lunhui Chang, Weiying Liu, Guorong Yan, Guolong Zhang

**Affiliations:** ^1^ School of Medicine, Anhui University of Science and Technology, Huainan, China; ^2^ Department of Phototherapy, Shanghai Skin Disease Hospital, School of Medicine, Tongji University, Shanghai, China; ^3^ Skin Cancer Center, Shanghai Skin Disease Hospital, School of Medicine, Tongji University, Shanghai, China; ^4^ Institute of Photomedicine, School of Medicine, Tongji University, Shanghai, China; ^5^ Department of Dermatology, Hunan Aerospace Hospital, Changsha, China

**Keywords:** actinic keratosis, cutaneous squamous cell carcinoma, progression, malignant transformation, skin cancers, non-melanoma skin cancers

## Abstract

Cutaneous squamous cell carcinoma (cSCC) is the second most common non-melanoma skin cancer, among which 82% arise from actinic keratosis (AK) characterized by lesions of epidermal keratinocyte dysplasia. It is of great significance to uncover the progression mechanisms from AK to cSCC, which will facilitate the early therapeutic intervention of AK before malignant transformation. Thus, more and more studies are trying to ascertain the potential transformation mechanisms through multi-omics, including genetics, transcriptomics, and epigenetics. In this review, we gave an overview of the specific biomarkers and signaling pathways that may be involved in the pathogenesis from AK to cSCC, pointing out future possible molecular therapies for the early intervention of AK and cSCC. We also discussed current interventions on AK and cSCC, together with future perspectives.

## 1 Introduction

Cutaneous squamous cell carcinoma (cSCC) is a malignant tumor originating from epidermal keratinocytes. It is one of the most commonly diagnosed non-melanoma skin cancers (NMSC), with its incidence gradually increasing worldwide in recent years, accounting for approximately 20% to 50% of all skin tumors (1, 2). In the United States, there are approximately 1.1 million new cases of cSCC annually, with an overall increase in incidence of 263% between 1976-1984 and 2000-2010 (3, 4). In western China, cSCC has the highest incidence rate among all skin cancers, at 29.4%, and this rate continues to rise annually by 2.6% (5). In European countries, it is estimated that by 2030, the incidence of cSCC will double (6). With a large number of cases, cSCC presents a significant burden on public health, with 3% to 7% of patients experiencing metastasis, leading to notable morbidity and mortality, thus imposing substantial societal public health burdens (3, 7, 8).

Actinic keratosis (AK) is a precancerous skin lesion caused by long-term chronic exposure to ultraviolet radiation (UVR), predominantly affecting sun-exposed areas such as the head and face, and commonly occurring in the elderly. AK carries a high risk of developing into cSCC and can present with multiple clinical and subclinical lesions simultaneously. The progression rate and probability of AK to cSCC vary among individuals, with an estimated annual progression rate of 0% to 0.075% per lesion (9). Among elderly patients with multiple AK lesions, the annual rate of progression to invasive cSCC is 0.6%, increasing to 2.57% four years after diagnosis (10).

The increasing incidence and mortality rates of cSCC have significant implications for public health. Understanding the malignant transformation from AK to cSCC is crucial for the prevention, early detection, and effective management strategies of cSCC. Additionally, a deeper understanding of the molecular mechanisms underlying the transition from AK to cSCC may facilitate the development of novel targets for the intervention of AK or cSCC.

## 2 Etiology, prevention, and early diagnosis of AK

The etiological factors of AK include environmental and individual factors. Environmental factors primarily refer to exposure to UVR, with the incidence of AK being positively correlated with cumulative UVR exposure (11, 12). Populations exposed to more sunlight (e.g., Australia and the United Kingdom) have a higher incidence of AK (13). UVR can induce cellular genetic mutations, chronic skin inflammation, and immune suppression, ultimately leading to abnormal proliferation of keratinocytes. The more sunlight the skin absorbs, the higher risk of AK (14). Five independent risk factors for AK are age (>60 years), gender (male), skin type, history of skin tumors, and outdoor work history (14). Individuals with fair skin (Fitzpatrick types I and II) are more prone to AK, while the incidence of AK is similar in Fitzpatrick types III and IV skin (12, 15). These conditions are influenced by various exposome factors such as air pollution, nutrition, and psychological stress, all of which can exacerbate skin carcinogenesis and aging (16). Additionally, immunosuppressed populations, such as 32.5% solid organ transplant recipients (SOTRs) (17) or patients taking long-term cytotoxic drugs, are at higher risk of AK (18–21).

Prevention of AK is strongly recommended, with evidence supporting using sunscreen and protective clothing to block initial and recurrent UVR radiation (22, 23). Randomized controlled trial (RCT) has shown that the use of sunscreen can reduce the incidence and progression of additional AK lesions (24). In addition to the significant role of sunscreen in AK prevention, oral medications also have a preventive effect on AK, such as oral nicotinamide (25–27), and acitretin (28, 29). However, a recent double-blind, randomized, placebo-controlled trial of oral nicotinamide for the prevention of AK in kidney transplant recipients showed that nicotinamide did not reduce the progression of AK in this population (30).

The diagnosis of AK emphasizes early detection and prediction of the risk of AK progression to cSCC. Non-invasive and highly efficient diagnostic techniques such as dermatoscopy, reflectance confocal microscopy (RCM), optical coherence tomography (OCT) (31), aminolevulinic acid (1%) (32), and high-frequency ultrasound of the skin (33) are employed for the diagnosis of AK. Dermatoscopy has good sensitivity and specificity for diagnosing AK, with a sensitivity of 95.6% and specificity of 95.0% for features such as the red pseudonetwork pattern combined with follicular pore enlargement (34). Dermatoscopic features of AK correlate with histological manifestations such as acanthosis, hyperkeratosis, and vascular proliferation. The “strawberry pattern” characteristic of non-pigmented AK can be detected by dermatoscopy and differentiated from early cSCC (35, 36). The most specific indications for diagnosing AK with RCM are the observation of disordered arrangements of keratinocyte layers and cellular atypia, with RCM having a unique diagnostic value for pigmented AK (12, 34). Histopathological examination remains the gold standard for diagnosing AK, especially when clinical diagnosis is uncertain or when progression to invasive cSCC is suspected. The main indications for pathological examination are uncertain clinical diagnosis, lesions > 1 cm in diameter, bleeding, ulceration, or induration, and rapid growth of skin lesions; secondary indications include lesions accompanied by severe itching, pain, and obvious hyperkeratosis (37). Skin lesions on certain special sites (such as the lips) are at high risk of invasion and require pathological examination. In addition, pathological subtyping helps guide the treatment and prognosis assessment of AK. Common pathological subtypes of AK include hypertrophic type, atrophic type, dyskeratotic type, mossy type, pigmented type, Bowenoid type, and actinic cheilitis type. Although AK histological subtypes have their characteristics, they mostly manifest as dysplastic keratinocytes arranged disorderly from mild basal layer to full-thickness epidermal structure disorder, appearing as a continuous spectrum structure of *in situ* squamous cell carcinoma, often accompanied by hyperkeratosis and dyskeratosis.

## 3 Etiology, prevention, and early diagnosis of cSCC

cSCC shares similar risk factors with AK, including exposure to UVR, age, fair skin, and immunosuppression (38). UVR exposure stands out as the primary risk factor for cSCC. Prolonged sun exposure, cumulative exposure in specific areas, and incidents of sunburn are closely correlated with cSCC incidence, particularly among individuals with fair skin (38). The *TP53* mutations induced by UVR represent the most common genetic alterations in cSCC (39). This malignancy is more prevalent among Caucasians, with an average onset age of approximately 60 years, and the risk increases with age, with a male-to-female ratio of about 3:1 (40). Immunocompromised individuals, such as SOTRs, face a significantly higher risk of developing cSCC, with SOTRs being 65 to 250 times more susceptible than healthy adults (41–43). Additionally, human papillomavirus (HPV) has been implicated in cSCC pathogenesis, particularly in cases affecting the perianal or genital regions (44). Environmental factors contributing to cSCC include arsenic exposure, polycyclic aromatic hydrocarbons, nitrosamines, and exposure to ionizing radiation (45, 46). Early signs of arsenic exposure include the occurrence of palmoplantar keratosis (45). Genodermatoses such as xeroderma pigmentosum, epidermodysplasia verruciformis, and epidermolysis bullosa dystrophica are also risk factors for cSCC development (47).

The “Interdisciplinary Guidelines for Invasive cSCC Based on European Consensus: Part 1 – Diagnosis and Prevention - 2023 Update” emphasizes primary and secondary prevention strategies for cSCC (48). Primary prevention involves avoiding sunbathing, using protective clothing, applying sunscreen, and seeking shade to minimize UVR exposure (48). Chemical prevention aims to reduce the incidence of new cSCC cases and includes agents such as nicotinamide, retinoids, 5-FU, and nonsteroidal anti-inflammatory drugs (NSAIDs). Nicotinamide can repair DNA damage and immunosuppression caused by intense UVR (49). However, a 12-month RCT involving SOTRs showed that oral nicotinamide did not reduce the incidence of cSCC (50). Several studies have demonstrated that oral retinoids, particularly acitretin and isotretinoin, can lower the incidence of new cSCC cases, especially among high-risk individuals (51, 52). An RCT on 5-FU showed that topical treatment reduced the expected surgical risk of cSCC by 75% (53). Population-based case-control studies suggest an association between NSAID use and a reduced risk of cSCC, with frequent NSAID use being a protective factor against cSCC development (54).

Digital cSCC lacks distinctive clinical features, leading to a potential misdiagnosis rate of up to 82% if solely based on clinical manifestations (55). Histopathological examination remains the gold standard for cSCC diagnosis, with well-differentiated histopathological subtypes exhibiting low metastatic potential, including keratoacanthoma and verrucous carcinoma, while aggressive subtypes associated with poor prognosis include desmoplastic cSCC, adenosquamous cSCC, and cSCC arising in association with scars (56, 57). Dermoscopy provides early diagnostic clues for cSCC and aids in the approximate assessment of its pathological staging, playing a crucial role in diagnosis, subsequent treatment, and management (58). cSCC lesions exhibit two vascular patterns under dermoscopy: dotted vessels and glomerular vessels. *In situ* pigmented cSCC may also present with small brown globules and homogeneous brown pigmentation under dermoscopy. Invasive cSCC often displays circular or serpentine vessels. RCM enables non-invasive diagnostic assessment of cSCC or AK skin lesions, with cSCC lesions showing a disordered arrangement of epidermal cells or atypical honeycomb patterns, along with circular vessels in the papillary dermis (59, 60).

## 4 Established progressed-associated genes from AK to cSCC

**Figure 1:**
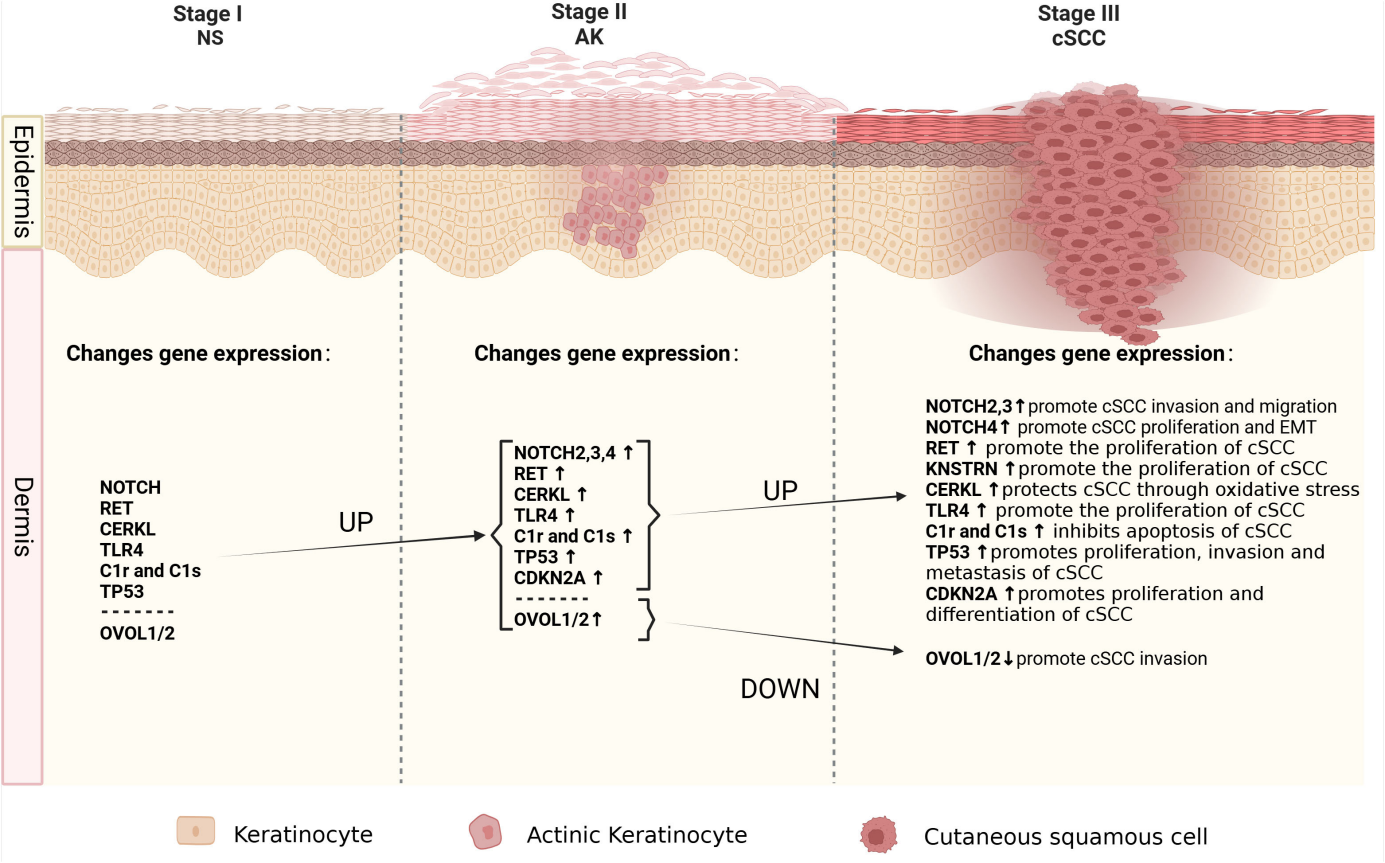
The molecular alterations associated with cancer progression from normal skin to AK to cSCC. Schematic depicting the spectrum of morphologies of NS-AK-cSCC lesions, with relevant changes in gene expression.

**Figure 2:**
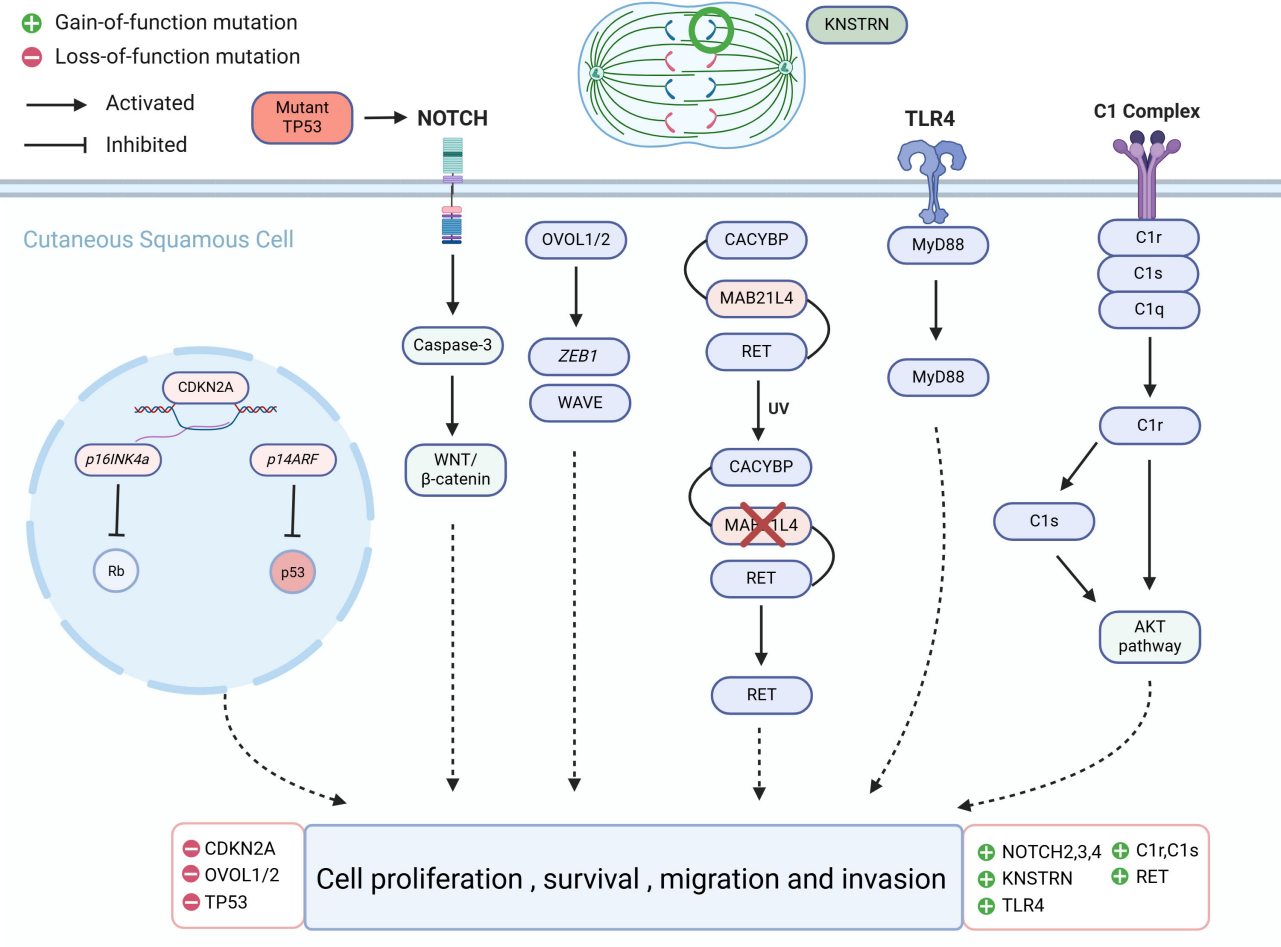
Molecular alterations that drive cutaneous squamous cell carcinoma (cSCC) proliferation.

Due to the fact that more than 70% of cSCC cases arise from AK (61), understanding the mechanisms underlying the progression from AK to cSCC is of paramount importance as it could aid in early therapeutic interventions before malignant transformation occurs. Identifying specific genomic changes that drive the transition from normal skin (NS) to AK and subsequently to invasive cSCC is challenging, given the substantial burden of UVR-induced mutations characteristic of all stages of this progression (**Figure 1**). Molecular genetic studies have examined AK and cSCC for known cancer gene expressions, chromosomal instability, and mutation levels (**Figure 2**). These studies generally indicate that AK and cSCC share similar genetic alterations (**Table 1**).

**Table 1:** The mutations and mechanisms in cSCC and AK genes across published studies.

Gene name	Gene Locus	Gene Function and Mechanism	Gene Alteration(from AK to cSCC)	Reference
TP53	Chr 17p	Regulating cellular processes such as DNA repair, cell cycle arrest, and apoptosis	Down	(62, 63)
TP63	Chr 3q28	Targeting for p38α signal transduction and regulating various cellular physiological processes, including cell proliferation, cell differentiation, inflammatory responses, and cell apoptosis	Down	(81, 84)
NOTCH1	Chr 9q34	Regulating upstream by TP53.Regulating downstream factors TP63, TP21, caspase-3, and the WNT/β-catenin pathway.	Down	(98, 100) (101, 104)
NOTCH2	Chr 1p13-p11		Up	
NOTCH3	Chr 19p13.2-p13.1		Up	
CDKN2A	Chr 9p21.3	Encoding two alternatively spliced proteins, p16INK4a and p14ARF.p16INK4a is associated with the β-catenin pathway. Inhibiting cell cycle progression and proliferation through the RB pathway and TP53 pathway	Down	(109, 110)
RET	Chr 10q11.2	Lossing of MAB21L4 and involving in the signaling pathways that regulate cell proliferation, differentiation, and migration	Up	(124)
OVOL1	Chr 11q	Promoting cSCC by inhibiting the expression of ZEB1 and WAVE.	Up	(138, 140)
OVOL2	Chr 20			
KNSTRN	Chr 15	Playing a role in cell division and the cell cycle.	Up	(143, 144)
CERKL	Chr 2q31.3	Promoting the development and progression of cSCC through oxidative stress.	Up	(147)
TLR4	Chr 9q	Recognizing and responding to exogenous PAMPs.Involving the production of cytokines, activation of the NF-κB signaling pathway, and the promotion of antibacterial and antiviral immune responses.	Up	(151, 153)
C1r and C1s	Chr 12p	Promoting the activation of ERK1/2 and Akt, inhibit apoptosis, and facilitate the growth and angiogenesis of cSCC.	Up	(158, 160)

### 4.1 TP53

The tumor protein p53 (*TP53*) is situated on the short arm of chromosome 17 and encodes a 53-kilodalton protein involved in regulating cellular processes such as DNA repair, cell cycle arrest, and apoptosis (62, 63). *TP53* is widely recognized as the most common mutated gene in cancer, being a pivotal tumor suppressor gene, whose abnormalities are associated with most malignancies (62). UVR-induced *TP53* mutations are early events in cSCC development, leading to genomic instability, followed by mutations in other tumor suppressor genes and oncogenes (64). The mutation frequency of *TP53* in primary cSCC ranges from 50% to 60%; in metastatic cSCC, *TP53* mutations are present in nearly 95% of patients (65, 66). *TP53* mutations are also present in normal skin exposed to UVR, AK, *in situ*, and invasive cSCC, indicating that *TP53* mutations are not only early manifestations of UVR damage but also associated with cSCC progression (65, 66). Targeted deep sequencing of 160 cancer-related genes in 17 AK and cSCC patients revealed driver mutations in *TP53* in 81% of cases, suggesting widespread *TP53* mutations in AK and cSCC lesions (67). *TP53* gene mutations have been found in a significant proportion of human skin malignancies, not only participate in the malignant transformation from AK to cSCC but also serving as biomarkers for predicting skin cancer (68). Skin cells exposed to ionizing radiation, such as solar UVR, incur DNA damage, leading to destabilization and inactivation of *TP53* (69). *TP53* gene mutations or alterations in the *TP53* signaling pathway during the progression from AK to cSCC result in functional impairment of *TP53*, shifting its role from tumor suppression to oncogenesis, manifested as clonal proliferation of keratinocytes. The most typical pattern in AK is CC>TT base changes in abnormally proliferating keratinocytes, leading to further genetic instability and eventual cSCC development (65, 70). This clonal proliferation is considered a critical step in cSCC development (71). A deep sequencing study of skin clonal mutations demonstrated that *TP53* R248W and G245D hotspot mutations are highly recurrent, potentially representing early stages of cSCC development (65).

Immunohistochemical results showed that the expression level of *TP53* in cSCC was slightly lower than that of AK, and the expression level was significantly lower than that of NS (72, 73). Missense mutations occur in *TP53* in cSCC, with common mutation hotspots such as TP53 R248W. Mutant *TP53* gains oncogenic activity, serving as a potent early driver of cSCC progression. Additionally, missense mutations of *TP53*, such as p53 R172H, can lead to metastatic cSCC development (74). Besides missense mutations in *TP53*, some mutations result in nonsense mutations, thereby prematurely introducing a termination codon (75). Truncated isoforms produced by TP53 nonsense mutations include tumor-specific expressions characterized by increased metastasis, which may be a feature of tumor metastasis (76, 77). The functional mechanisms of *TP53* mutant variants, including activation of cell proliferation, promotion of cell invasion and metastasis, and the presence and potential functions of different *TP53* subtypes in cSCC development, are not yet fully understood.

Transcription factor (TF) *TP63* is a p53 family member and plays a key role in the development of keratinocyte transformation induced by basal cell and ectodermal appendage carcinoma genes in stratified cSCC cell survival (78–82). p63 is a major regulator of epidermal development and maintenance and is also a key target for *p38α* signal transduction (81). The level of p63 protein detected in a large number of human cancer cell lines is negatively correlated with phosphorylated p38 signaling (81). *P38α* is an important signal-transducing protein kinase that belongs to the mitogen-activated protein kinase (MAPK) family (83). It plays a role in regulating various cellular physiological processes, including cell proliferation, cell differentiation, inflammatory responses, and cell apoptosis (79–81, 84). In cSCC biopsies, a significant reduction in p38 phosphorylation in the tumor parenchyma links AK with SCC progression and p38 inactivity (85). p38α plays a role in the transition from AK to cSCC, and p38α kinase is involved in the suppression of cSCC by regulating p63 and stem cell frequency (85).

*TP53*, as a pivotal gene, holds significant clinical relevance and applications in AK and cSCC. *TP53* mutations are found in up to 100% of AK cases and 90% of cSCC cases, indicating its central role in the pathogenesis of these skin lesions (86). *TP53* mutations are an early event that leads to the inactivation of its tumor suppressor function, facilitating the accumulation of additional oncogenic mutations and progression from AK to invasive cSCC (65). These mutations often result in a loss of normal p53 function and can lead to more aggressive tumor phenotypes (87). *TP53* serves as a diagnostic and prognostic biomarker, the presence of *TP53* mutations can serve as a diagnostic marker for AK and cSCC, as well as a prognostic indicator of disease progression and potential resistance to therapy (88). Reduced p53 staining in AK is associated with a higher likelihood of progression to cSCC, particularly in common and hypertrophic types of AK (86). Additionally, strategies to target *TP53* mutations include the development of small molecules that reactivate mutant p53 or prevent its degradation, thereby restoring its tumor suppressor function (89). These approaches are promising in enhancing the efficacy of existing treatments and overcoming resistance in *TP53*-mutated tumors (90). However, not all *TP53* mutations have the same biological impact. The loss of E-cadherin and increased expression of p53 are associated with the progression of AK to cSCC (91). The loss of E-cadherin and increased p53 are associated with AK progression, but it is important to consider the broader context of these molecular changes (91). While *TP53* mutations are a hallmark of AK and cSCC, their role extends beyond tumor suppression to influencing therapeutic outcomes. The development of targeted therapies that address the specific mutations and pathways involved in *TP53* dysfunction holds promise for improving patient outcomes. However, the complexity and variability of *TP53* mutations necessitate a nuanced approach to treatment and research.

### 4.2 NOTCH

The NOTCH signaling pathway serves as a convergence point for numerous essential signaling pathways and is one of the most critical pathways determining cell fate. Signal transduction between adjacent cells via *NOTCH* receptors regulates cell differentiation, proliferation, and apoptosis, playing a widespread role in the occurrence and development of malignant tumors. Loss-of-function mutations in *NOTCH1*, *NOTCH2*, and *NOTCH3* are commonly found in cSCC and sun-exposed skin, including AK, which does not exhibit microscopically evident dysplasia (66). *NOTCH1*, *NOTCH2*, and *NOTCH3* genes are located on chromosomes 9q34, 1p13-p11, 19p13.2-p13.1, respectively (92). *NOTCH* signaling maintains the homeostasis of keratinocytes, and in AK, *NOTCH1* mutations can occur in extracellular or transmembrane domains, as well as in intracellular domains. These mutations can disrupt the cellular microenvironment signaling both inside and outside the cell, leading to a predisposition to cancer (93–97). A genomic profiling study of AK revealed that *NOTCH1* and *NOTCH2* are significantly mutated genes, with mutation levels positively correlated with UVR intensity (98, 99). This study also found that *NOTCH1* mRNA is significantly upregulated in AK compared to healthy skin, suggesting that *NOTCH1* may have a carcinogenic role in AK, contrary to its presumed tumor-suppressive role in cSCC, where *NOTCH1* is typically inactivated (98, 100). Further research is needed to elucidate the distinct functional roles of *NOTCH1* in AK and cSCC progression.

Approximately 40% of cSCC patients exhibit inactivating mutations in *NOTCH1*, making it the second most common mutated gene after *TP53* (101, 102). Studies indicate upregulation of *NOTCH1* expression in cSCC, suggesting that *NOTCH1* may function as both a tumor suppressor and a facilitator, exerting both tumor-suppressive and tumorigenic effects in cSCC (101). *NOTCH2*, *NOTCH3*, and *NOTCH4* may act as oncogenes in cSCC, with studies showing high expression of *NOTCH2* and *NOTCH3* in cSCC tumor tissues. Upregulation of *NOTCH2* expression may promote tumor cell migration and invasion, while high expression of *NOTCH3* may be positively correlated with tumor cell proliferation and volume (101). *NOTCH4* is upregulated in cSCC through the NOTCH4/HEY1 pathway, inducing tumor cell proliferation, resistance to cisplatin, and promoting epithelial-to-mesenchymal transition (EMT) (103). The expression of *NOTCH* genes is associated with upstream gene *TP53* and downstream regulatory factors *TP63*, *TP21*, *caspase3*, and the WNT/β-catenin pathway, although the specific mechanisms remain unclear (66, 101).

The clinical application of targeting the *NOTCH* gene in AK and cSCC involves understanding its dual role as both an oncogene and a tumor suppressor, depending on the cellular context (104). This dual role complicates the therapeutic targeting of *NOTCH*, as its inhibition could potentially have opposing effects depending on the specific cancer type and stage (105). Gamma-secretase inhibitors (GSIs) are a class of drugs that inhibit the activation of *NOTCH* receptors by preventing the cleavage of the *NOTCH* intracellular domain (106). Furthermore, given the complexity of *NOTCH* signaling, combination therapies that target multiple pathways are being investigated. For instance, combining *NOTCH* inhibitors with other signaling pathway inhibitors, such as those targeting the PI3K/Akt or VEGF pathways, may enhance therapeutic efficacy (105). These inhibitors are being explored in clinical trials for their potential to suppress tumor growth in cancers with aberrant *NOTCH* signaling (107). Nevertheless, inhibiting *NOTCH* signaling can have unintended consequences due to its role in normal cellular processes (104). Therefore, careful consideration of the potential side effects and the development of selective inhibitors are crucial for successful clinical application.

### 4.3 CDKN2A

The *CDKN2A* gene is situated on chromosome 9p21.3, a region known for its involvement in tumorigenesis across different cancer types (108). It encodes two alternatively spliced proteins, *p16INK4a* and *p14ARF*, which inhibit cell cycle progression and proliferation through the RB pathway and *TP53* pathway, respectively (98, 109, 110). When *CDKN2A* is highly expressed, it inhibits the proliferation of tumor cells. However, mutations or inactivation of *CDKN2A* can diminish its ability to suppress tumor cells, leading to rapid proliferation and differentiation (111). Exome sequencing studies of AK have identified *CDKN2A* as a non-candidate driver gene (98). Previous research also suggests that *CDKN2A* does not mutate in normal skin and may serve as a gatekeeper in the AK-cSCC transition (112). Genomic studies of somatic mutations and copy number alterations in AK and cSCC have found that *CDKN2A* driver mutations are more common in cSCC (30.8%) than in AK (11.1%), indicating fewer genetic mutations in *CDKN2A* in AK. However, *CDKN2A* is located on chromosome band 9p21, one of the most common genomic alteration regions in human cancers (113). Loss of heterozygosity at 9p21, leading to CDKN2A mutations or homozygous deletion, results in loss of function, which may contribute to the AK-to-cSCC transition (109, 114). Mutation analysis of *CDKN2A* loci in 26 AK specimens revealed new mutations, additional mutations, and missense mutations, corresponding to four different nucleotide changes, three in exon 1a of *p16INK4a* and one in exon 2 (115).

*CDKN2A* is strongly associated with the occurrence of skin malignancies. As a tumor suppressor gene, *CDKN2A* negatively regulates the cell cycle and promotes cellular senescence. Knockout mouse experiments suggest that *p14ARF* may play a role in the initiation of cSCC development, while *p16INK4a* may be involved in determining the differentiation status of cSCC (109, 116). Recurrent *CDKN2A* mutations have been found in skin malignancies, and inactivation of *CDKN2A* at the locus may be due to allelic loss, point mutations, and promoter hypermethylation. Inactivation of the *CDKN2A* locus leads to loss of control over the cell cycle and cell growth (117). Heterozygous loss or point mutations have been detected in 21% to 62% of cSCC cases, and promoter hypermethylation has been found in 35% to 78% of cases. Recently, immunohistochemical analysis of cSCC precursor lesions revealed a linear correlation between the immunostaining of *p16INK4a* and the membranous immunostaining of β-catenin in AK, indicating the importance of *p16INK4a* in the progression of cSCC precursor lesions (118).

*CDKN2A* mutation and expression levels serve as valuable prognostic markers and potential therapeutic targets. *CDKN2A* mutations are significantly associated with disease-specific death in metastatic cSCC, indicating its potential as a prognostic biomarker. Patients with *CDKN2A* mutations exhibit shorter disease-specific survival compared to those without such mutations (119). In cSCC, *CDKN2A* mutations are early genetic events, often occurring alongside *TP53* mutations (119). These mutations are present in both primary tumors and metastases, suggesting their role in the early stages of tumorigenesis. The presence of *CDKN2A* mutations correlates with adverse clinical outcomes, such as increased tumor size and invasion depth, which are significant predictors of poor survival in cSCC patients (119). In the context of *CDKN2A*-associated therapeutic approaches, *CDKN2A* expression is linked to immune cell infiltration and immune regulation pathways, which could influence the tumor microenvironment and response to immunotherapy (120). The gene’s role in immune regulation and its association with immune checkpoints like PD-1 suggest that *CDKN2A* could guide immunotherapy strategies, potentially improving responses in cancers where it is highly expressed (121). The gene’s ability to influence tumor progression and response to therapies highlights its importance as a therapeutic target and biomarker. However, further research is needed to explore its specific applications in AK and cSCC, considering the unique pathophysiology of these skin conditions.

### 4.4 RET

*RET* (Rearranged during transfection) is located on chromosome 10q11.2 and is involved in the signaling pathways that regulate cell proliferation, differentiation, and migration (122). Through different splicing mechanisms, *RET* can undergo 3′-end cleavage to form various receptor tyrosine kinases, including *RET9*, *RET43*, and *RET51*, which constitute the single-pass transmembrane tyrosine kinase receptor protein RET family. These isoforms exhibit tissue-specific expression (123). The exact role of *RET* in AK and cSCC remains unclear. It is activated by binding with soluble glial cell-derived neurotrophic factor family ligand proteins, leading to the production of *RET* proteins with abnormal activity. These aberrant signals may influence processes such as cell growth, survival, invasion, and metastasis. In AK, *RET* is significantly expressed and can drive cSCC, and the activation mechanism of *RET* is associated with the loss of *MAB21L4* (124). *MAB21L4*, also known as Male Abnormal 21-like 4 or C2orf54, plays a role in differentiating human progenitor epidermal keratinocytes (125). *MAB21L4* is considered a bridging protein between *CACYBP* and *RET*. In the presence of *MAB21L4*, the binding affinity between *CACYBP* and *RET* increases by 44 times. *CACYBP* can bind to the E3 ubiquitin ligase SIAH1 and participate in various cellular processes, including cell differentiation, proliferation, cytoskeletal dynamics, and tumorigenesis (126). High expression of *RET* in AK is caused by the loss of *MAB21L4*, which is an early event in AK. This loss disrupts the proximity of *RET* to the CACYBP-SIAH1 complex, preventing *RET* ubiquitination (124).

While the primary focus of *RET* inhibitors has been on lung and thyroid cancers, their application in skin cancers like AK and cSCC is an area of interest. The application of *RET* inhibitors in treating these cancers has evolved significantly, with a focus on developing selective inhibitors to improve efficacy and reduce side effects. This approach is particularly relevant in the context of AK and cSCC, where targeted therapies are increasingly being explored (127). The development of selective *RET* inhibitors like selpercatinib and pralsetinib has marked a significant advancement in targeting *RET*-driven cancers (128). These inhibitors have shown high efficacy and favorable toxicity profiles compared to earlier multikinase inhibitors (MKIs), which had off-target effects and limited clinical activity (129). Selpercatinib and pralsetinib have been approved by the FDA for treating *RET*-altered cancers, demonstrating high response rates in clinical trials (130). These inhibitors are effective against *RET* gatekeeper mutations, which are common resistance mechanisms in cancer therapy (131). The exploration of *RET* inhibitors in skin cancers is still in its early stages. However, the success of these inhibitors in other cancer types suggests potential applicability, especially in cases where *RET* alterations are identified (132).

### 4.5 OVOL1/2

*OVOL1* and *OVOL2* (*OVOL1/2*) belong to the OVO gene family and are located on chromosomes 11 and 20, encoding a class of DNA-binding proteins that typically function as transcription factors regulating gene expression, acting as both transcriptional activators and repressors (84, 133–136). Three members of the OVO gene family have been identified: *OVOL1*, *OVOL2*, and *OVOL3* (137). Among them, (*OVOL1/2*) have been studied most extensively. *OVOL1/2* participate in suppressing EMT in tumor cells (133). They can inhibit the expression of Zinc finger *E-box binding homeobox 1* (*ZEB1*) in tumor cells (138). *OVOL1* levels are significantly elevated in non-metastatic oral squamous cell carcinoma tissue and negatively correlated with *ZEB1* levels. *OVOL2* is significantly downregulated in metastatic nasopharyngeal carcinoma cells, which exhibit strong invasive capabilities. Knockdown of *ZEB1* can reduce the invasion capability of these cells (139). In 30 AK and 30 cSCC cells, knocking down *OVOL1/2* increased the mRNA expression of *ZEB1* and WAVE proteins. Specifically, knocking down *OVOL1* significantly increased the expression of *ZEB1* and WAVE proteins, while knocking down *OVOL2* significantly increased the expression of ZEB1. Loss of *OVOL2* significantly enhanced the invasion capability of cSCC cells (140). Immunohistochemical analysis of AK and cSCC cells showed that the expression of *OVOL1* and *OVOL2* was upregulated in AK but significantly downregulated in cSCC. Conversely, *ZEB1* and WAVE proteins were upregulated in cSCC, indicating that the downregulation of *OVOL1/2* and the upregulation of *ZEB1* and WAVE proteins may be associated with the progression of AK to cSCC (140).

The application of *OVOL1* and *OVOL2* genes in the treatment of AK and cSCC is a promising area of research due to their roles in modulating EMT. This makes them attractive targets for therapeutic strategies aimed at halting or reversing the progression of these skin lesions. Targeting the *OVOL2/ZEB1* axis could be a viable therapeutic approach to prevent the development of cSCC from AK. By inhibiting *ZEB1*, *OVOL2* can potentially reduce the invasiveness of cSCC cells, making it a promising target for drug development (140). While the potential of *OVOL1* and *OVOL2* in treating AK and cSCC is promising, it is important to consider the complexity of cancer progression and the multitude of genetic and environmental factors involved. Therefore, while targeting *OVOL1* and *OVOL2* could be beneficial, a comprehensive approach that considers these other factors may be necessary for effective treatment.

### 4.6 KNSTRN

*KNSTRN* is a protein that binds to Astrin on the kinetochore in the late stage of cell mitosis and is closely related to cell division (141). The *KNSTRN* gene is located on chromosome 15 at the 15q15.1 locus. It encodes a protein that is essential for proper chromosome alignment and segregation during cell division, particularly mitosis (142). *KNSTRN* is believed to play a role in cell division and the cell cycle process. Recurrent mutations were detected in 19% of AK and cSCC, mainly concentrated in *KNSTRN* mutations (143). Exome sequencing of 112 cSCC genes showed that the mutation sites were located at the N-terminus. 21 out of 112 (18.7%) patients had somatic mutations in *KNSTRN*, with over half of the mutations occurring at codon 24, resulting in a change from serine to phenylalanine due to a C-T transition (143). Detection of the frequency of *KNSTRN* gene mutations in healthy skin, photo-damaged skin, AK, and invasive cSCC with different histological grades showed that recurrent somatic mutations in *KNSTRN* p.Ala40Glu were associated with basal proliferative AK in invasive squamous cell carcinoma (144). These studies also suggest that *KNSTRN* may play a driving role in the occurrence and development of cSCC and could be a useful predictor for cSCC development.

The *KNSTRN* gene has emerged as a significant player in the development and progression of AK and cSCC. Mutations in this gene, particularly the recurrent somatic mutations, have been linked to the acceleration of cSCC development and are associated with different histological classifications of AK. These findings suggest that targeting the *KNSTRN* gene could be a promising strategy for treating these skin conditions. Given the role of *KNSTRN* mutations in cSCC progression, therapies targeting these mutations could potentially halt or reverse the disease process. This could involve the development of small molecules or biologics that specifically inhibit the mutated forms of the *KNSTRN* protein (140). Techniques such as CRISPR-Cas9 could be employed to correct *KNSTRN* mutations in affected cells, potentially preventing the progression of AK to cSCC (140). While *KNSTRN* mutations are prevalent in cSCC, they are not exclusive to this cancer type, as they have also been found in other cutaneous tumors like malignant melanomas (140). This necessitates a careful approach to ensure that therapies targeting *KNSTRN* mutations do not adversely affect normal tissues.

### 4.7 CERKL

The equations should be inserted in editable format from the equation editor. *Ceramide kinase-like* (*CERKL*) is situated on chromosome 2q31.3, and it is an oxidative stress-related protein whose expression is typically limited to the retina, nervous tissue, kidneys, trachea, testes, and lungs (145, 146). Levels of *CERKL* in normal skin are relatively low, but *CERKL* mRNA and protein are highly expressed in AK and cSCC (147). Knocking down *CERKL* in cSCC cells reduces the content of cell sheath lipids and enhances the sensitivity of cSCC cells to oxidative stress, indicating that *CERKL* may protect cSCC cells through an oxidative stress protection mechanism (147). The mechanism of oxidative stress protection mediated by *CERKL* is currently unclear but it may involve *CERKL*-mediated regulation of cellular autophagy (148). The mechanisms of *CERKL* in AK and cSCC require further investigation.

Studies have identified various genes involved in cSCC progression, including *CERKL*, which could serve as biomarkers for early diagnosis and treatment strategies (149). *CERKL* is highly expressed in cSCC and AK compared to normal epidermis. It plays a role in maintaining cellular sphingolipids and protecting cells from oxidative stress, which are critical in the survival and proliferation of cancer cells (149). By enhancing resistance to oxidative stress, *CERKL* may contribute to the survival of cancer cells under hostile conditions, making it a potential target for therapies aimed at increasing cancer cell susceptibility to oxidative damage (149). While *CERKL* presents a promising target for cSCC and AK treatment, it is essential to consider the complex genetic and molecular landscape of these conditions.

### 4.8 TLR4

Toll-like receptor 4 (*TLR4*) is located on chromosome 9q in humans, and it also is a receptor protein in the human immune system (150). It belongs to the Toll-like receptors (*TLRs*) family and plays a major role in regulating immune responses and inflammatory responses. TLR4 protein is mainly expressed on the surface of immune cells such as macrophages, dendritic cells, and certain epithelial cells. Its main function is to recognize and respond to pathogen-associated molecular patterns (PAMPs) from exogenous pathogens. *TLR4* binds to these PAMPs and activates immune cells, triggering a series of immune responses, including the production of cytokines, activation of the NF-κB signaling pathway, and promotion of antibacterial and antiviral immune responses. *TLR4* is expressed in human skin, AK, and cSCC (151). *TLR4* signal transduction promotes cSCC development in a MyD88-dependent manner and is essential for inflammatory cell recruitment during carcinogenesis (152). *TLR4* may play a tumor-promoting role in cSCC, and immunohistochemical detection shows that the expression of *TLR4* in cSCC is significantly higher than that in AK tissue, and its expression in low-differentiated cSCC is significantly lower than that in moderately differentiated and highly differentiated cSCC (153).

The clinical application of the *TLR4* gene in AK and cSCC involves understanding its role in tumor progression and immune response modulation. *TLR4* antagonists, such as eritoran and resatorvid, have been identified as potential therapeutics. These agents can suppress UV-induced inflammatory signaling, which is crucial in preventing skin photodamage and photocarcinogenesis (154). On top of that, TLR agonists have been used in immunotherapy for skin cancers, including melanoma and basal cell carcinoma, by enhancing dendritic cell recruitment and T-cell responses (155). This approach could be adapted for cSCC, targeting *TLR4* to modulate immune responses (156).

### 4.9 C1r, C1s, and factor D

*C1r* and *C1s* are two important components of the complement system, which belong to the *C1* complex. Both C1r and C1s genes are located on chromosome 12, specifically in the region 12p13 (157). The *C1* complex is the initial enzyme complex of the complement system and participates in the regulation of immune responses and inflammatory reactions. *C1r* and *C1s* are both enzyme proteins that play a key role in the *C1* complex. *C1r* plays the role of an initiator enzyme in the activation of the *C1* complex. When *C1r* is activated, it catalyzes the activation of *C1s* (158). *C1s*, on the other hand, is responsible for further activating other components of the complement system, such as *C4* and *C2* (159). Compared with normal epidermal keratinocytes, the mRNA levels and production of C1r and C1s are significantly increased in cSCC cells (160). Reducing the expression of *C1r* and *C1s* in cSCC cells suppressed the activation of extracellular signal-regulated kinase 1/2 and Akt, promoting cSCC cell apoptosis and significantly inhibiting the growth and angiogenesis of human cSCC xenografts (158). Furthermore, *C1r* and *C1s* were highly expressed in aggressive disseminated cSCC and cSCC associated with pemphigus vulgaris, while they were expressed less in *in situ* cSCC, AK, and normal skin (160).

Given their role in promoting cSCC growth, *C1r* and *C1s* represent potential targets for clinical application therapeutic intervention. As markers of aggressive tumor behavior, *C1r* and *C1s* could help predict the likelihood of progression from AK to invasive cSCC, aiding in risk stratification and management decisions (160). Inhibitors or monoclonal antibodies against these proteins could be developed to suppress tumor growth and improve patient outcomes (160). The integration of *C1r* and *C1s* targeting into clinical practice will require further validation in larger cohorts and the development of specific inhibitors.

Complement factor D(FD) cleaves C3 to produce the active fragments C3a and C3b. C3a can induce inflammatory reactions, causing vasodilation and promoting the infiltration of inflammatory cells (161–163). In culture and *in vivo*, cSCC cells express FD significantly. The *in vivo* FD expression level in non-metastatic cSCC, metastatic cSCC, cSCC metastasis, and pemphigus vulgaris-related cSCC is significantly higher than that in normal skin, precancerous lesions, AK, and *in situ* cSCC. The strongest FD expression is limited to the invasive edge of the tumor, while the weak FD staining pattern in normal skin, AK, and cSCC *in situ* shows a diffuse and uniform distribution (164).

FD’s role in the alternative complement pathway makes it a promising target for therapeutic intervention. The small-molecule FD inhibitor Danicopan has been proposed as a specific drug candidate for advanced cSCC therapy (164). Inhibition of FD activity has been shown to attenuate cSCC cell proliferation, suggesting that targeting FD could be an effective strategy for managing cSCC progression (164). While the role of FD is promising in the treatment of cSCC, further research is needed to fully elucidate the complex interactions between these factors and to validate FD’s clinical utility in larger, diverse patient populations.

## 5 Nomenclature established progressed-associated non-coding RNAs from AK to cSCC

RNA, as an integral part of gene regulation, has garnered widespread attention, with non-coding RNA (ncRNA) emerging as a focal point of research in recent years. Variants such as long non-coding RNA (lncRNA) and microRNA (miRNA) have been particularly emphasized. The precise mechanisms and specific targets of ncRNA are still actively under investigation, as they serve as potential regulatory molecules with significant roles in skin cancer. Further research endeavors will contribute to a better understanding of the exact functions of RNA molecules in the progression from AK to cSCC and their potential clinical applications.

### 5.1 Long non-coding RNA

Long non-coding RNA (lncRNA) refers to a group of endogenous RNAs with lengths exceeding 200 nucleotides, lacking complete open reading frames, and thus not encoding proteins (165). However, lncRNA can interact with DNA, RNA, and proteins through various mechanisms, participating in related biological processes such as serving as protein scaffolds, directly interacting with proteins and RNA (166), participating in chromatin modification, regulating mRNA, transcription factors, and epigenetics, and acting as competing endogenous RNA for microRNA (167). A study employing RNA sequencing of two cases of AK and three cases of normal skin tissue identified lncRNA uc011fnr.2 negatively regulating *SCIMP* and *TLR4*, and being involved in activating the JAK-STAT3 signaling pathway in AK (168). Additionally, the dysregulation of lncRNAs in premalignant lesions like actinic keratosis (AK) suggests their involvement in the progression to invasive cSCC (168).

### 5.2 miRNA

*miRNAs* are short non-coding RNAs that regulate protein-coding gene expression at the post-transcriptional level. *miRNAs* play important roles in the initiation and progression of tumorigenesis by regulating the expression of tumor suppressor genes and oncogenes (169–171). The most significant changes in *miRNA* expression occur during the transition from “early” stages of normal epidermis, AK, and early photodamaged epidermis to the “late” stages of epidermal carcinogenesis, late-stage AK, and cSCC (172). By analyzing the differential expression of genes in 10 AK and 30 cSCC tissue samples, using microarray dataset GSE45216 obtained from the gene expression database, they identified 16 miRNAs and found that three co-expressed network modules *EIF4EBP1*, *SNX17*, *PRPF4*, *NXT1*, and *UBA5* may be pathogenic genes contributing to the development of AK into cSCC (173). Recent studies have also demonstrated that miRNA regulation plays a critical role in the progression of epidermal keratinocytic neoplasms (174, 175).

*miR-204* primarily functions as a tumor suppressor gene. It has been shown that DNA methylation in the upstream promoter region of TRPM3, a gene upstream of *miR-204*, is one of the mechanisms contributing to the silencing of *miR-204* in cSCC (176). Comparison of *miR-204* expression between cSCC, AK, and healthy skin tissues, revealed significantly downregulated *miR-204* in cSCC. Overexpression of *miR-204* inhibits the activation and translocation of transcription activator 3, thereby suppressing the development of cSCC. Protein tyrosine phosphatase non-receptor type 11 (PTPN11) has been identified as a direct target of *miR-204* (176). *miR-23b* is downregulated in both AK and cSCC, and its expression is regulated by the MAPK signaling pathway. *miR-23b* reduces the expression of FGF2 at both mRNA and protein levels, impairing the ability of cSCC cells to induce angiogenesis. Overexpression of miR-23b inhibits colony and sphere formation in cSCC cells, while CRISPR/Cas-9-mediated deletion of miR-23b leads to increased colony and tumor sphere formation *in vitro* (177). Experimental studies have confirmed the overexpression of Ras-related protein RRAS2 in cSCC, and the interference of RRAS2 expression impairs angiogenesis, colony formation, and tumor sphere formation. Importantly, RRAS2 is a direct target of *miR-23b* in cSCC (177). miR-181a acts as a transcriptional driver factor in the progression from AK to cSCC. *miR-181a* promotes the formation of various proteins by targeting the TGFβ receptor 3 (TGFβ R3) in the TGFβ signaling pathway. In cSCC, the expression level of *miR-181a* is inversely correlated with the expression level of TGFβ R3. Overexpression of *miR-181a* or knockdown of TGFβ R3 inhibits apoptosis and enhances cell survival. Moreover, miR-181a enhances cell migration and invasion and upregulates epithelial-mesenchymal transition markers. *miR-181a* regulates these processes by directly interacting with TGFβ R3. Experimental results have demonstrated that *miR-181a* inhibits tumor growth, and reversing these effects can be achieved by increasing or decreasing TGFβ R3 expression (178). Additionally, a study has shown the involvement of *miRNA-31* and MMP-1 in differentiation pathways associated with increased EMT and angiogenesis, which are important mechanisms in cSCC invasion. EMT is enhanced in AK (179). These findings hold significant implications for future research on targeted therapies.

## 6 Application of clinical drugs in AK and cSCC

**Figure 3:**
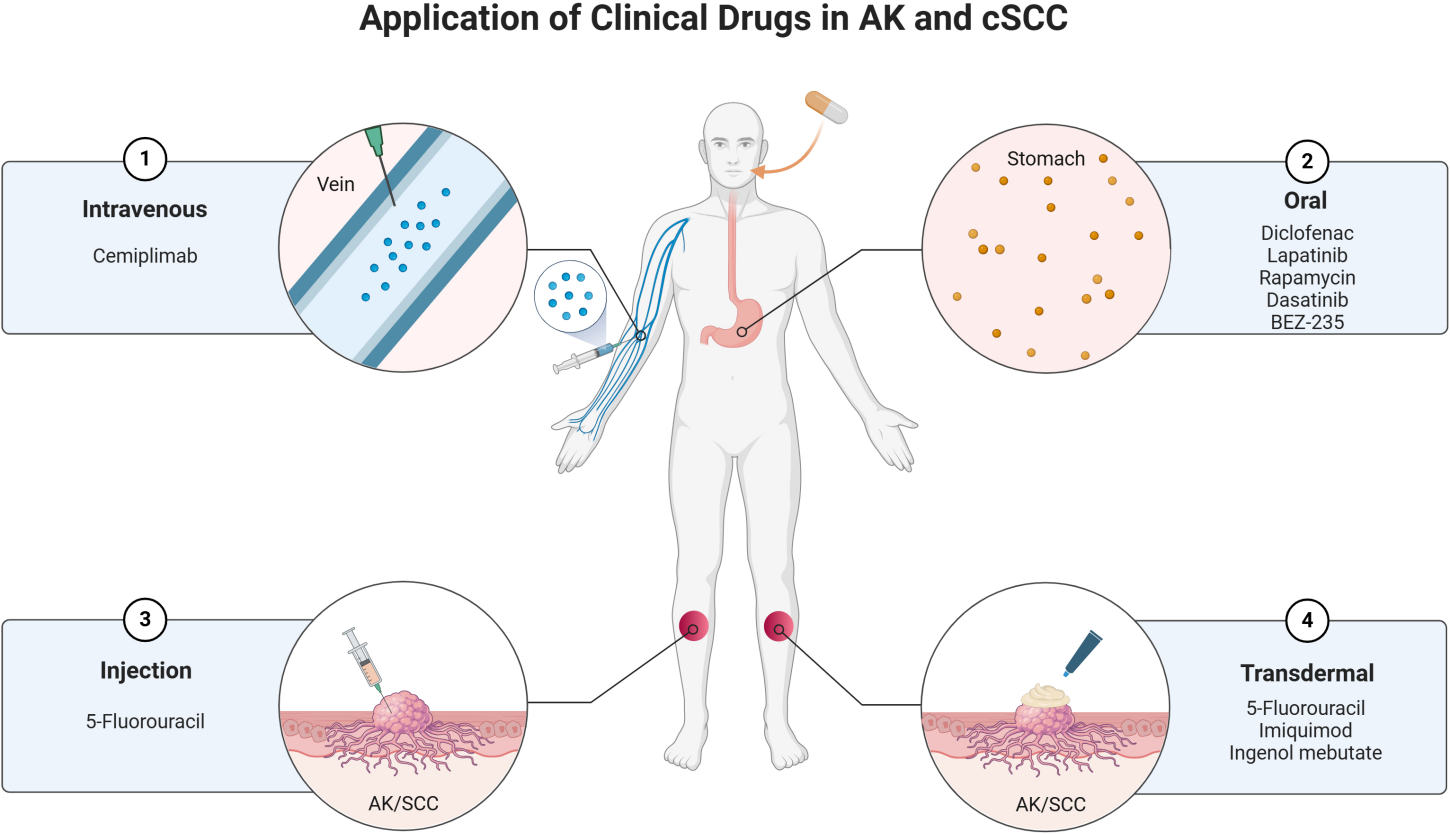
Current clinical drugs used in the management of AK and cSCC.

The treatment of AK and cSCC involves a variety of clinical drugs and therapeutic approaches. These conditions, which are closely related, often require a combination of topical, systemic, and procedural treatments to manage effectively. The choice of treatment depends on factors such as the severity of the lesions, patient characteristics, and the potential for malignant transformation. Below is an overview of the current clinical drugs used in the management of AK and cSCC (**Figure 3**).

### 6.1 Topical treatments

#### 6.1.1 5-fluorouracil

This is a widely used topical chemotherapeutic agent for AK and cSCC (180). A novel 4% formulation of 5-FU cream has shown high efficacy in treating multiple AKs, with significant reductions in the Actinic Keratosis Area and Severity Index (AKASI) scores observed at 6 and 12 weeks post-treatment (181). It works by inhibiting DNA synthesis in rapidly dividing cells, leading to the eradication of AK and cSCC lesions (182). Combining 5-FU with photodynamic therapy (PDT) has been shown to enhance lesion clearance rates (183). Pretreatment with 5-FU increases protoporphyrin IX accumulation, improving the efficacy of PDT in AK treatment (183). Unlike its use in the treatment of AK, 5-FU has alternative approaches for managing cSCC. Intralesional 5-FU at lower concentrations (10.0 mg/mL and 16.7 mg/mL) has been effective in treating cSCC and keratoacanthomas, achieving a 96% lesion clearance rate with minimal adverse effects (184). This approach strategically reduces the cumulative dose of 5-FU, thereby mitigating potential toxicities without compromising therapeutic efficacy. Furthermore, a combinative treatment strategy involving debulking surgery followed by intralesional 5-FU has evinced robust tumor clearance rates, particularly in instances of well-differentiated SCCs (185). This integrated method has proven especially efficacious for managing tumors located on the trunk and extremities, offering a tailored and effective treatment modality for such cases. While 5-FU has proven effective in treating AK and cSCC, its application requires careful consideration. The potential for combination therapies, such as with PDT, offers promising avenues for enhanced treatment outcomes. However, the variability in response rates and the need for individualized treatment plans underscore the complexity of managing these conditions. Further research is needed to optimize 5-FU use across diverse patient groups and to explore its full potential in preventing progression to invasive cSCC.

#### 6.1.2 Imiquimod

Imiquimod is widely used for AK treatment due to its ability to modulate the immune system, leading to the clearance of lesions. This immune response modifier is used in 5% cream form for AK treatment (186). It works by inducing cytokine production, which promotes apoptosis in abnormal keratinocytes (187). Studies have shown clearance rates ranging from 66% to 87% when used as monotherapy (188). However, combining imiquimod with other agents like 5-fluorouracil has been explored to enhance efficacy (188). This combination has shown higher clearance rates and shorter treatment durations, suggesting a synergistic effect (188). In the treatment of cSCC, imiquimod has been used off-label for treating cSCC, particularly in cases where surgery or radiation is not feasible (189). A notable case involved the successful treatment of a locally advanced cSCC of the eyelid with imiquimod 3.75%, resulting in complete tumor regression without significant side effects (189). While imiquimod has demonstrated efficacy in treating AK and cSCC, its application is not without challenges. The variability in patient response, potential for local skin reactions, and the need for further studies to confirm long-term outcomes and optimal treatment regimens are areas that require attention. Overall, imiquimod remains a valuable tool in dermatological oncology, with ongoing research likely to expand its utility and effectiveness.

#### 6.1.3 Diclofenac

Diclofenac, a non-steroidal anti-inflammatory drug (NSAID), has been explored for its application in treating AK and cSCC. Diclofenac’s mechanism involves the inhibition of cyclooxygenase-2 (COX-2), reducing prostaglandin E2 levels, which are implicated in tumorigenesis and inflammation associated with AK (190). And the drug also induces apoptosis and alters cell cycle profiles in cancerous cells, potentially through p53 induction and metabolic alterations, as observed in esophageal squamous cell carcinoma models (191). Diclofenac 3% gel, often combined with hyaluronic acid, is a topical treatment for AK. It has been shown to be effective, with a resolution of at least 50% of lesions in 78% of patients, and a high satisfaction rate of 85% (192). But in a comparative study, diclofenac was less effective than imiquimod in preventing the progression of AK to more severe forms or invasive SCC over a three-year period (192) While diclofenac offers a viable option for treating AK and potentially cSCC, its role is often complementary to other therapies. Its ability to modulate immune responses and cellular metabolism presents a unique advantage, but its efficacy in preventing progression to invasive SCC is less than some alternatives. Future research may focus on optimizing its use in combination therapies and exploring its full potential in cancer prevention and treatment.

#### 6.1.4 Ingenol mebutate

Ingenol mebutate is FDA-approved for treating AK, a precursor to cSCC (193). It induces cell death through the PKC/MEK/ERK signaling pathway, leading to reduced cell viability and proliferation in keratinocyte-derived cells (194). Despite its efficacy, there are concerns about an increased risk of SCC in patients treated with ingenol mebutate for AK, as indicated by pharmacovigilance studies (195). Ingenol mebutate exhibits diverse therapeutic applications; however, its association with an increased risk of SCC and adverse reactions in certain treatments underscores the necessity for cautious application and further research to ensure safe and effective use.

### 6.2 Systemic treatments

#### 6.2.1 Cemiplimab

Cemiplimab, a monoclonal antibody targeting the programmed cell death protein 1 (PD-1), has demonstrated high efficacy in treating advanced cSCC, with objective response rates (ORR) ranging from 32% to 77% in various studies (196). In a phase II trial, cemiplimab showed a 62% ORR with a complete response in 22% of patients (197). Real-world data indicate a clinical benefit rate of 54.3% and a median progression-free survival of 12.2 months (198). While cemiplimab has shown significant efficacy in treating advanced cSCC, its application in AK is less documented in the provided contexts. The focus remains on cSCC, where cemiplimab has established itself as a critical therapeutic option.

#### 6.2.2 Lapatinib

Lapatinib, a tyrosine kinase inhibitor, has shown potential in the treatment of cutaneous squamous cell carcinoma cSCC and AK by targeting the EGFR and HER2 pathways (199). It disrupts the PI3K/AKT/mTOR signaling pathway, leading to cell cycle arrest in the G2/M phase and suppression of EMT in cSCC cells (200). A phase 2 clinical trial demonstrated that short-term lapatinib treatment resulted in tumor size reduction in 2 out of 8 patients with cSCC (199). Additionally, a mean regression of 30% in AK lesions was observed after 56 days of treatment (199). While lapatinib shows promise in treating cSCC and AK, its efficacy as a monotherapy is limited, and side effects such as hepatotoxicity pose challenges.

#### 6.2.3 mTOR inhibitors

mTOR inhibitors like rapamycin work by blocking the mTOR pathway, which is crucial for cell growth and proliferation (201). This blockade leads to decreased phosphorylation of mTOR targets, resulting in apoptotic death of cancer cells and reduced growth and metabolic activity of surviving cells (201). Inhibition of mTOR also reduces the expression of mutant p53, a common mutation in SCC, further contributing to the reduction of tumor growth (201). Oral administration of mTOR inhibitors in UVB-induced mouse models has been effective in reducing the number and size of AK and cSCC lesions, indicating their potential in both treatment and prevention of these conditions (202). While mTOR inhibitors show significant promise in the treatment and prevention of AK and cSCC, there are challenges and considerations to address. Additionally, while preclinical models provide valuable insights, clinical trials are essential to confirm efficacy and safety in humans. The potential for topical formulations of mTOR inhibitors could mitigate systemic side effects and offer a more targeted approach, but further research is needed to optimize these treatments (203).

### 6.3 Emerging therapies

Agents like dasatinib and BEZ-235, these small molecule kinase inhibitors have shown efficacy in inducing regression of cSCC with minimal inflammation and side effects (180). They target kinases that promote keratinocyte growth, offering a promising therapeutic approach for AK and cSCC (180). The development of topical kinase inhibitors represents a significant advancement in dermatological therapeutics, particularly for conditions like AK and cSCC. These inhibitors provide a targeted approach that minimizes systemic exposure and associated risks, making them a valuable addition to the treatment landscape. However, it is important to consider the potential for adverse effects and the need for further research to optimize their use in clinical practice.

## 7 Current limitations and future perspectives

The progression from AK to cSCC is a multifactorial process influenced by genetic, environmental, and molecular mechanisms, with approximately in the progression from AK to cSCC, a comprehensive understanding of the precise molecular mechanisms remains limited, largely due to the complex interplay between genetic, epigenetic, and environmental factors. The identification of potential biomarkers for early detection and prognosis of cSCC remains a challenge, with many findings yet to be validated in clinical settings. Current therapeutic interventions for AK and cSCC are limited, with few targeted therapies available, particularly due to the heterogeneity of genetic mutations in key genes such as *TP53* and *NOTCH*. Additionally, there is a scarcity of clinical trials focusing on early intervention in AK to prevent its progression to cSCC, with most trials concentrated on advanced stages of cSCC. The significant variability in progression rates among individuals, influenced by genetic predisposition, immune status, and environmental factors, further complicates the establishment of standardized treatment protocols. Integrating multi-omics data (e.g., genomics, transcriptomics, and proteomics) into a coherent framework to understand the progression from AK to cSCC remains a formidable challenge.

In the future, multi-omics approaches, integrating genomics, transcriptomics, and proteomics, can reveal complex molecular mechanisms and identify potential biomarkers for AK. This can aid in understanding the progression from AK to SCC and in developing targeted interventions (204). By analyzing the unique molecular profile of each patient, multi-omics can facilitate personalized treatment plans, potentially improving efficacy and reducing adverse effects. Implementing personalized treatment strategies based on patient genetic profiling may enhance therapeutic efficacy. There is an urgent need for more clinical trials focusing on the early stages of AK and evaluating the effectiveness of preventive strategies, including combination therapies targeting multiple pathways simultaneously. Public health initiatives should promote awareness and preventive measures against UV exposure to reduce the incidence of AK and cSCC, emphasizing education about sun protection and early skin assessments for early detection. Furthermore, further studies on the roles of non-coding RNAs, such as lncRNAs and miRNAs, in the progression from AK to cSCC may reveal new regulatory mechanisms and potential therapeutic targets.

## 8 Discussion

The progression from AK to cSCC is a multifactorial process influenced by genetic, environmental, and molecular mechanisms, with approximately 70% of cSCC cases originating from AK (205). Key drivers include chronic UVR exposure, which induces *TP53* mutations, a frequent early event in the AK-to-cSCC pathway (206). *TP53* mutations disrupt cell cycle regulation, DNA repair, and apoptosis, contributing to genomic instability and abnormal keratinocyte proliferation (207). Identifying *TP53* mutations in normal skin exposed to UVR indicates their potential as biomarkers for early risk stratification and targeted prevention strategies, emphasizing the importance of genetic screening and early intervention to reduce cSCC burden (208).

In addition to *TP53*, genes such as *NOTCH*, *CDKN2A*, and *RET* play critical roles in the AK-to-cSCC transition. The *NOTCH* pathway, involved in cell differentiation and proliferation, exhibits dual functions as both a tumor suppressor and promoter in AK and cSCC, with mutations disrupting keratinocyte homeostasis, offering therapeutic potential (209). *CDKN2A* mutations contribute to the loss of cell cycle control, underscoring the importance of genomic integrity (210). Furthermore, dysregulation of lncRNAs and miRNAs influences gene expression and cellular behavior, with miR-204 acting as a tumor suppressor and miR-181a promoting survival and invasion, highlighting their roles as therapeutic targets in cSCC progression (176, 178).

Moreover, current treatments for AK and cSCC, such as topical agents like 5-fluorouracil and imiquimod, exhibit varied efficacy. Incorporating molecular markers and genetic profiling into clinical practice could optimize therapeutic selection and enhance patient outcomes, with specific *TP53* mutations potentially benefiting from targeted therapies addressing underlying genetic alterations. Additionally, emerging systemic treatments like cemiplimab and mTOR inhibitors show promise in advanced cSCC, while topical kinase inhibitors provide a novel, minimally systemic approach to treating both AK and cSCC, potentially reducing adverse effects of traditional therapies.

Despite the progress made in understanding the molecular mechanisms underlying the transition from AK to cSCC, several challenges remain. The heterogeneity of tumors, variations in individual responses to UVR, and the influence of host factors complicate the development of universal treatment strategies. Moreover, the potential for resistance to therapies targeting specific mutations highlights the need for personalized approaches in managing patients with AK and cSCC.

## 9 Conclusion

cSCC remains a major public health challenge, and the progression from AK to cSCC in normal skin is still not well understood. However, the progression of AK to cSCC represents an example of the transformation of a precursor lesion into an aggressive malignancy. The comprehensive review of the development mechanisms from AK to cSCC has provided valuable insights into the complex processes underlying this progression. By deepening our knowledge and understanding of the underlying mechanisms, we can advance the development of targeted prevention strategies, early detection methods, and personalized therapeutic approaches. Ultimately, these efforts will contribute to reducing the incidence, improving prognoses, and enhancing the overall management of cSCC.
